# A Comparison Between Phone-Based Psychotherapy With and Without Text Messaging Support In Between Sessions for Crisis Patients

**DOI:** 10.2196/jmir.3096

**Published:** 2014-10-08

**Authors:** Gareth Furber, Gabrielle Margaret Jones, David Healey, Niranjan Bidargaddi

**Affiliations:** ^1^Health Economics and Social Policy GroupSchool of Population HealthUniversity of South AustraliaAdelaideAustralia; ^2^Mental Health Observatory Research UnitSchool of MedicineThe University of AdelaideAdelaideAustralia; ^3^IAPT@Flinders unitSouthern Mental HealthFlinders Medical CentreAdelaideAustralia; ^4^Mental Health Informatics Research UnitCountry Health SA LHN IncAdelaideAustralia; ^5^e-Health ResearchSchool of MedicineFlinders UniversityAdelaideAustralia

**Keywords:** telemedicine, psychotherapy, mental health services, mobile health, mHealth, short message service, eHealth

## Abstract

**Background:**

Few studies have tested whether individually tailored text messaging interventions have an effect on clinical outcomes when used to supplement traditional psychotherapy. This is despite the potential to improve outcomes through symptom monitoring, prompts for between-session activities, and psychoeducation.

**Objective:**

The intent of the study was to explore the use of individually tailored between-session text messaging, or short message service (SMS), as an adjunct to telephone-based psychotherapy for consumers who present to the Emergency Department (ED) in situational and/or emotional crises.

**Methods:**

Over a 4-month period, two therapists offered 68 prospective consumers of a telephone-based psychotherapy service individually tailored between-session text messaging alongside their telephone-based psychotherapy. Attendance and clinical outcomes (depression, anxiety, functional impairment) of those receiving messages were compared against a historical control group (n=157) who received telephone psychotherapy only.

**Results:**

A total of 66% (45/68) of the consumers offered SMS accepted the intervention. A total of 432 messages were sent over the course of the trial, the majority involving some kind of psychoeducation or reminders to engage in therapy goals. There were no significant differences in clinical outcomes between consumers who received the SMS and those in the control group. There was a trend for participants in the intervention group to attend fewer sessions than those in the control group (mean 3.7, SD 1.9 vs mean 4.4, SD 2.3).

**Conclusions:**

Both groups showed significant improvement over time. Individually tailored SMS were not found to improve clinical outcomes in consumers receiving telephone-based psychotherapy, but the study was underpowered, given the effect sizes noted and the significance level chosen. Given the ease of implementation and positive feedback from therapists and clients, individually tailored text messages should be explored further in future trials with a focus on enhancing the clinical impact of the tailored text messages, and utilizing designs with additional power to test for between-group effects.

## Introduction

Multiple authors have discussed the value of mobile phones in the treatment of mental disorders [1-8]. The characteristics of mobile phones—popularity, widespread use, low cost, portable, always on, advanced multimedia capabilities, can run complex apps—mean there are many avenues for mental health professionals to harness their potential in treatment. Despite the significant potential, only a small number of studies have tested whether supplementing mental health care with mobile phone-based interventions leads to better consumer outcomes.

Three studies have shown that symptom monitoring alongside usual care can lead to better outcomes. Spaniel and colleagues [9] used Short Message Service (SMS), or text messaging, for symptom monitoring to identify prodromal symptoms in patients with schizophrenia and alert psychiatrists to initiate medication changes. The addition of SMS monitoring to their usual case management was shown to reduce the number of hospitalizations and hospitalization length. Kramer et al [10] used a system of mobile phone symptom monitoring and clinical feedback in patients receiving medication for depression. Patients who were monitored and provided feedback showed a larger decrease in depression symptoms over 6-month follow-up, compared to both a control (no monitoring) and pseudo-control (monitoring but no feedback). Reid, Kauer, and colleagues [11,12] found that a daily mobile phone mental health assessment and feedback program delivered through primary care to young people with mental health problems, led to improvements in emotional self-awareness and depression.

Two studies have shown that the use of text message reminders alongside usual care can improve treatment processes. Pijnenborg et al [13] used daily text message reminders as a form of “cognitive aid” to assist patients with schizophrenia in achieving treatment goals (eg, attend appointments, take their medication). When receiving reminders, patients showed a significant increase in percentage of goals achieved. Similarly, Montes and colleagues [14] showed that daily text message medication reminders for patients with schizophrenia increased medication adherence compared to those who did not receive reminders.

Finally, three studies have shown that delivering mobile phone-based aftercare can improve post-treatment outcomes. Bauer and colleagues [15] found that a text messaging-based symptom reporting and tailored feedback system delivered post-treatment to women with eating disorders led to increased remission rates at 8 months post-treatment. Agyapong et al [16] found twice-daily supportive/educational text messages relating to mood and alcohol abstinence led to lower depression scores at 3 months post-discharge in patients who had recently completed an in-patient dual diagnosis (depression and alcohol use) treatment program. Marasinghe and colleagues [17] demonstrated that a brief mobile phone-based treatment involving training and advice on problem solving, social support, and drugs and alcohol, delivered to patients following hospitalization for a suicide attempt, led to lower rates of suicidal ideation and depression at 6 and 12 months post-hospitalization.

In all of these examples, mobile phones were used to supplement standard care (ie, case management, primary care, medication). The evidence that mobile phone apps can improve outcomes of psychotherapy is less convincing. Case studies and small uncontrolled trials have demonstrated that mobile devices (including mobile phones) can feasibly be used as part of psychotherapy such as cognitive behavior therapy (CBT). For example, personal digital assistants (PDA), precursors to modern smartphones, have been used to assist in the cognitive behavioral treatment of obsessive compulsive disorder [18], schizophrenia [19], social phobia [20], bulimia nervosa [21], and generalized anxiety disorder [22]. More recently, Aguilera et al [23] used text messaging to send participants attending a group CBT program, daily messages regarding mood monitoring and content of group sessions. Participants reported the messages made them feel closer to the group and more likely to attend group sessions. Rizvi and colleagues have also piloted the use of a mobile phone app to complement dialectical behavior treatment for borderline personality disorder [24]. However, none of these studies used control groups to determine if the addition of the mobile phone improved consumer outcomes.

The goal of the current study was to develop an individualized text messaging support intervention as an adjunct to an existing telephone-based psychotherapy service and evaluate it using a historical control group. The setting for this study was a small team of psychotherapists who provide telephone-based psychotherapy to consumers who recently presented to the Emergency Department (ED) in situational and/or emotional crises. To our knowledge, the value of supplementing psychotherapy in this population with tailored text messaging interventions has not been explored.

We developed a text messaging intervention where therapists and consumers collaborate to compose goal reminder and psychoeducation messages, individually tailored to the consumer’s goals, and deliver these messages between sessions. This was in response to emerging evidence that individuals are sensitive to variations in the linguistic content of text messages designed to help them achieve a personal goal [25]. Tailoring text message content to an individual can be more effective compared to automated pre-determined messages [26]. The choice to develop a text messaging intervention (rather than a mobile phone app) was made because text messaging is widely available on mobile phones, has an extremely low learning curve, and requires minimally expensive and complex infrastructure. We also noted that the majority of work in the area, as described above, has used text messaging, and that reviews of text messaging interventions in the wider health literature (eg, [27-29]) have suggested significant value in their use.

## Methods

### Setting

The current project was conducted within the “IAPT@Flinders” service. IAPT@Flinders is an Australian outgrowth of the United Kingdom’s Improving Access to Psychological Therapies (IAPT) model, which provides rapid access to evidence-based, low intensity psychological interventions, based on CBT principles for people suffering mild to moderate depression and anxiety [30]. IAPT@Flinders targets consumers who present to the ED in situational and/or emotional crises who are not subsequently admitted to psychiatric services. The fundamental goal of the IAPT@ Flinders service is to provide an alternative evidence-based rapid access treatment pathway for these consumers, and thus reduce the burden on ED of mental health crisis readmissions. Inclusion criteria for the service include: medical clearance to leave the ED, aged 18 years and over, not currently engaged with a mental health service or currently receiving psychological therapy, and experiencing strong psychological distress including anxiety and/or depressive symptoms.

The service provided to IAPT@Flinders consumers includes low intensity telephone-based CBT as well as referral and linkages to other key community services (eg, relationship, drug and alcohol, employment, housing, or financial services). The low-intensity CBT treatment component consists of providing consumers with self-help resources relevant to their presenting symptoms and supporting them in working through these materials. The self-help resources (eg, [31]) typically include psychoeducation about depression and/or anxiety and a range of cognitive and behavioral strategies such as behavioral activation, cognitive restructuring, exposure therapy, sleep and panic management, and motivational problem solving. The telephone sessions involve explaining key CBT principles and supporting consumers in setting goals based on the techniques described in the self-help materials. A unique aspect of the IAPT program that facilitates evaluation is the use of session-by-session measures of outcome. This means any consumer attending at least two appointments will have two data points.

### Study Design and Participants

We hypothesized that consumers who received individually tailored text messaging in addition to their psychotherapy would show improved clinical outcomes (levels of depression, anxiety, and functional impairment) and increased attendance compared to those receiving psychotherapy alone.

Two IAPT@Flinders therapists from a pool of four volunteered to take part in the trial after it was described to them by the authors (GF, GJ, NB). Over a 4-month period in 2013, these two therapists offered all consumers presenting to the service the opportunity to receive individually tailored text messages alongside their usual care. For a consumer to be offered the text messaging, they needed to (1) meet criteria for treatment with IAPT@Flinders, (2) attend the initial assessment (telephone or face-to-face), (3) consent to receive text messages, and (4) own and be able to use a mobile phone that could receive text messages. Consumers declining the text messaging intervention were not evaluated further. The sample was considered a self-selected sample.

The impact of individually tailored text messaging was assessed by comparing attendance and clinical outcomes of those receiving the text messages (intervention group) to a historical control group. The choice to use a historical control group versus control group by randomization was pragmatic, reflecting multiple barriers to implementing randomization in the service, including resources, personnel, risk of consumer disengagement, and contamination.

### Intervention Group

The intervention group received individually tailored text messages alongside their usual care. The text messaging intervention was developed collaboratively by IAPT@Flinders clinicians and the IAPT@Flinders research group (GF, GJ, NB), and was designed to complement the goal setting already being conducted with consumers. The intervention was designed to be simple to implement, low cost, and involve minimal extra clinician time. Two basic message types were conceptualized. The first (reminders) were specific prompts to engage in activities/homework discussed in session. For example, consumers were reminded to engage in physical activity if they had set that as a goal for the week. The second (psychoeducation) were reiterations of key concepts discussed during therapy. For example, consumers were reminded of recommended methods for managing feelings of anxiety such as slow breathing or reassurance that feelings of anxiety will pass naturally.

Participants were introduced to the intervention as follows. While in treatment and at the end of each session, the therapist would ask the consumer if it would be useful to send some text message reminders and psychoeducation during the upcoming week/fortnight in relation to what was discussed during the session. If the consumer consented, the therapist and consumer would collaboratively plan the content and schedule of those messages. As such, all messages were uniquely tailored to the consumer’s goals for that week/fortnight and the consumer was free to choose whether to receive text messages between each session.

Text messages were sent one-way and their delivery was coordinated by a Web-based program supplied by goACT Pty Ltd that enabled therapists to schedule messages for individual consumers, save message “templates” for use across different consumers, and to share between therapists. The content and schedule of messages was decided on a session-by-session basis and programmed into the system by the therapist at the end of the session. When reviewing consumers’ progress toward goals set in the previous week/fortnight, therapists inquired about the usefulness of the text messages in the previous week/fortnight.

Two safety protocols were developed for the study. The first required that all messages include the name of the therapist and the service: “Hi (first name of consumer), (first name of therapist) from IAPT here”. This was done to ensure consumers knew who the messages were coming from. The second protocol instructed therapists to cease scheduled messages (ie, delete them from the program) should it become apparent that the consumer had been re-hospitalized for self-harm or suicide attempt. This was to reduce the likelihood of the consumer being sent inappropriate messages during a crisis episode.

### Historical Control Group

Attendance and clinical outcomes of consumers receiving the text messaging intervention were compared to a historical control group. This group was made up of consumers who had received a service from IAPT@Flinders (from the same two therapists) in the 6 months prior to the introduction of the text messaging intervention. Data for the control group was extracted anonymously from the electronic clinical record. Similar to the criteria for the intervention group, only those who (1) met criteria for treatment with IAPT@Flinders, and (2) attended the initial assessment (telephone or face-to-face) and at least one other session were included in the historical control group. The control group consisted of 157 consumers.

### Measures

At intake to the service, all consumers provided their age, gender, employment status (employed, unemployed), education (high school complete: yes/no), relationship status (in a relationship: yes/no), prescribed psychotropic medication (yes/no), and whether they had a chronic health condition (yes/no).

Attendance was measured by recording treatment length (in days), whether treatment was completed, the number of telephone and face-to-face sessions that a consumer attended, as well as the number of recorded “did not attend” (DNA) sessions.

Clinical outcomes were measured using the 9-item Patient Health Questionnaire (PHQ-9), the 7-item Generalized Anxiety Disorder questionnaire (GAD-7), and the Work and Social Adjustment Scale (WSAS). For all consumers, the “pre” and “post” clinical outcomes corresponded to the first and last recorded instances of the three measures during their episode of care. On average, the time between the first and last recorded measures was 6 weeks.

The PHQ-9 is a 9-item self-reported questionnaire designed to evaluate the presence of depressive symptoms during the prior 2 weeks [32]. Each of the 9 items, relating to each of the DSM-IV diagnostic criteria for depression, can be scored from 0 (not at all) to 3 (nearly every day). In IAPT@Flinders, the PHQ-9 is used as a severity measure, with scores ranging from 0 (absence of depressive symptoms) to 27 (severe depressive symptoms).

The GAD-7 is a 7-item self-reported questionnaire designed to assess the presence of the symptoms of Generalized Anxiety Disorder (GAD), as listed in the DSM-IV [33]. The total GAD-7 score is calculated by summing the scores for all 7 items, which can be scored from 0 (Not at all) to 3 (nearly every day). In IAPT@Flinders, the GAD-7 is used as a severity measure, with scores ranging from 0 (minimal) to 21 (serious).

The WSAS is a 5-item self-reported questionnaire that assesses the degree of functional impairment attributable to an identified problem [34]. The WSAS score is calculated by summing the scores for all 5 items, which range from 0 (not at all) to 8 (severely). In IAPT@Flinders, the WSAS is used as an indicator of impairment with scores ranging from 0 (no impairment) to 40 (severe impairment).

### Analysis

*T* tests (continuous variables) and chi-square tests (categorical variables) were used to test for differences between the intervention and control groups on measures taken at intake and measures of attendance. Repeated measures analysis of variance (ANOVA) was used to compare the intervention and control groups on change in clinical outcomes (PHQ, GAD, and WSAS) over time. Analyses were conducted in SPSS Version 19.

## Results

**Figure 1:**
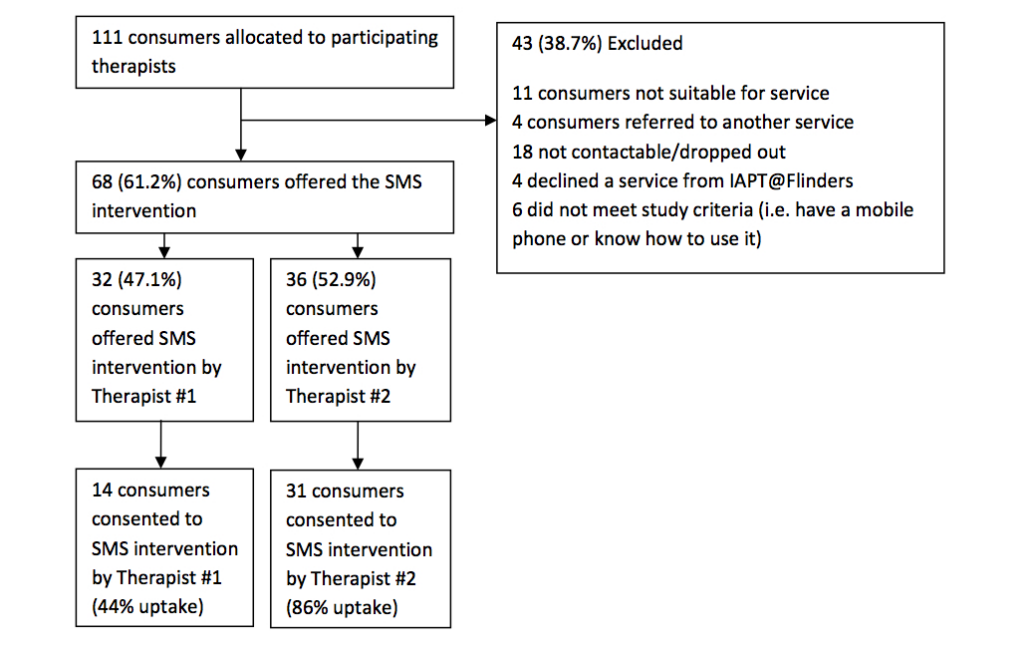
Flow of participants into the intervention group.

**Figure 2:**
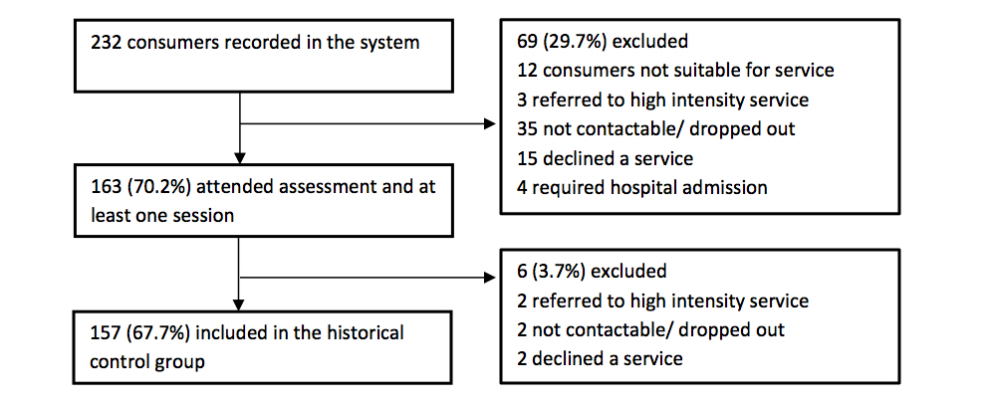
Flow of participants into the historical control group.

Of the 111 consumers allocated to the two participating therapists during the study period, 68 were offered the text messaging intervention. Reasons for exclusion are outlined in Figure 1. Therapist #2 was successful in recruiting 86% (31/36) of 36 consumers offered the intervention, in contrast with a consent rate of 44% (14/32) achieved by Therapist #1. In total, 45 consumers consented to and received the text messaging intervention alongside their usual IAPT@Flinders care. The historical control group was made up of 157 of the 232 clients recorded in the system at the time of analysis (Figure 2).

Intake status, attendance, and clinical outcomes for the intervention and control groups are summarized in Table 1. On a number of indicators, the intervention group appeared to be functioning better at intake: more were employed, more were in a relationship, fewer were prescribed psychotropic medication, and fewer reported a chronic health condition. Only one of these differences was statistically significant—consumers in the intervention group were less likely to be prescribed a psychotropic medication. Intervention and control groups did not differ significantly on their intake PHQ, GAD, or WSAS scores, suggesting similar levels of psychological distress.

Consumers in the intervention group were sent a total of 432 messages during the trial, consisting of 150 unique messages. Of these 150, 39 messages were sent two or more times, with the remainder being sent once only. The bulk of message content related to psychoeducation (eg, “Anxiety is normal and it will pass with time. Remember to slow your breathing when you start to feel anxious”), and reminders to engage in discussed homework tasks (eg, “Remember to schedule and complete behavioral activation tasks”).

Therapist #2 was responsible for the bulk of the messages sent (87.7%, 378/431). On average, consumers were sent 9.6 messages each, although there was wide variation. Over 90% (94.2%, 406/431) of messages were sent within 4 weeks of the consumers commencing therapy, with over 60% (61.9%, 267/431) sent in the first two weeks. Consistent with this, therapists reported that consumers tended to find the messages useful early in therapy, and less useful over time.

**Figure 3:**
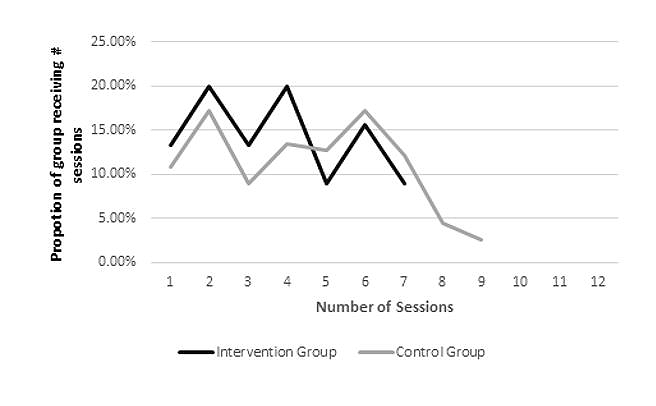
Proportion of intervention and control participants by number of sessions attended.

Regarding attendance, there were no statistically significant differences between intervention and control groups on treatment completion, treatment length, number of sessions attended, or DNA rate. A trend (*P*=.063) was observed for intervention participants to attend fewer sessions (−.7) than control participants. To explore this further, we charted the proportion of intervention and control groups by number of sessions received (Figure 3). This revealed that a greater proportion of intervention participants received 1-4 sessions whereas a greater proportion of control participants received 5-12 sessions.

Regarding clinical outcomes, there was a non-significant main effect of group on PHQ scores, *F*_1,199_=0.090, *P*=.764, partial η2=0.000, indicating intervention and control groups did not differ in their overall level of depressive symptomatology. There was a significant main effect of time on PHQ scores, *F*_1,199_=336, *P*=.000, partial η2=0.629, indicating both groups showed improvements in depressive symptomatology over time. There was a non-significant interaction between group and time on PHQ scores, *F*_1,199_=1.065, *P*=.303, partial η2=0.005, indicating no differences between groups in how depressive symptomatology changed over time.

There was a non-significant main effect of group on GAD scores, *F*_1,199_=0.508, *P*=.477, partial η2=0.003, indicating intervention and control groups did not differ in their level of anxiety symptomatology. There was a significant main effect of time on GAD scores, *F*_1,199_=283, *P*=.000, partial η2=0.587, indicating both groups showed improvements in anxiety symptomatology over time. There was a non-significant interaction between group and time on GAD scores, *F*_1,199_=2.346, *P*=.127, partial η2=0.012, indicating no differences between groups in how anxiety symptomatology changed over time.

There was a non-significant main effect of group on WSAS scores, *F*_1,199_=0.135, *P*=.713, partial η2=0.001, indicating intervention and control groups did not differ in their level of functional impairment. There was a significant main effect of time on WSAS scores, *F*_1,199_=199, *P*=.000, partial η2=0.500, indicating both groups showed improvements in functional impairment over time. There was a non-significant interaction between group and time on WSAS scores, *F*_1,199_=199, *P*=.892, partial η2=0.004, indicating no differences between groups in how functional impairment scores changed over time.

Additional ANOVA were conducted in which only those participants seen by Therapist #2 were included and where prescribed medication was included as a covariate. The pattern of results for PHQ, GAD, and WSAS did not change under these alternative models.

To determine if the study was adequately powered to detect differences between groups on attendance and clinical outcomes, we used G-Power [35] to conduct two post-hoc power analyses for the outcomes of number of sessions attended and change in PHQ scores over time (Table 2). These analyses revealed the probability of rejecting the null hypothesis, that the intervention and control groups were the same, was low for both number of sessions attended (.499), and change in PHQ scores over time (.378). Analyses of differences between groups should therefore be treated with caution.

**Table 1:** Intake status, attendance, and clinical outcomes for the intervention and control groups.

		Intervention group (n=45)	Control group (n=157)	Difference (95% CI)	Comparisons (*t* test, Pearson chi-square)	*P* value
**Intake status**						
	Age at assessment, mean (SD)	34.1 (14.5)	37 (15.8)	−3.51 (−8.7 to 1.67)	*t*_197_=−1.34	*P=*.183
	Gender, n (%) male	15 (33.3)	68 (43.3)	−10%	χ^2^_1_=1.44	*P*=.230
	Employment, n (%) employed	22 (48.9)	68 (43.3)	5.6%	χ^2^_1_=0.44	*P*=.507
	Relationship, n (%) yes	18 (40.0)	44 (28.0)	12%	χ^2^_1_=2.12	*P*=.145
	High school complete, n (%) yes	20 (44.4)	64 (40.8)	3.6%	χ^2^_1_=2.28	*P*=.131
	Prescribed psychotropic medication, n (%) yes	24 (53.3)	113 (72)	−18.7%	χ^2^_1_=6.02	*P*=.014
	Chronic health condition, n (%) yes	13 (28.9)	55 (35.0)	−6.1%	χ^2^_1_=0.44	*P*=.509
**Attendance**						
	Treatment completers, n (%)	28 (62.2)	111 (70.7)	−8.5%	χ^2^_1_=1.17	*P*=.279
	Treatment length in days, mean (SD)	43.3 (17.1)	46.3 (26.7)	−3.05 (−11.74 to 5.64)	*t*_194_=−0.89	*P*=.376
	Number of sessions, mean (SD)	3.73 (1.9)	4.43 (2.3)	−0.700 (−1.44 to 0.04)	*t*_200_=−1.87	*P*=.063
	Number of DNA^a^, mean (SD)	1.42 (1.12)	1.76 (1.51)	−0.342 (−0.82 to 0.14)	*t*_200_=−1.66	*P*=.100
**Clinical outcomes**						
	PHQ^b^Pre, mean (SD)	19.4 (6.4)	18.9 (5.6)	0.480 (−1.46 to 2.42)	*t*_200_=0.49	*P*=.626
	GAD^c^Pre, mean (SD)	16.1 (5.2)	15.8 (4.4)	0.350 (−1.18 to 1.88)	*t*_200_=0.45	*P*=.653
	WSAS^d^Pre, mean (SD)	27.7 (9.8)	27.0 (10.0)	0.730 (−0.26 to 4.06)	*t*_200_=0.43	*P*=.666
	PHQ Post, mean (SD)	6.64 (6.23)	7.57 (6.85)			
	GAD Post, mean (SD)	5.41 (5.51)	6.81 (6.17)			
	WSAS Post, mean (SD)	10.89 (10.70)	12.49 (12.44)			

^a^DNA: did not attend

^b^PHQ: Patient Health Questionnaire

^c^GAD: Generalized Anxiety Disorder questionnaire

^d^WSAS: Work and Social Adjustment Scale

**Table 2:** Post hoc power analyses for number of sessions attended and PHQ^a^scores.

	Number of sessions attended	PHQ scores
**Test**	*t* tests / Means: difference between two independent means (two groups)	*F* tests / ANOVA: Repeated measures, within-between interaction
**Analysis**	Post hoc: Compute achieved power	Post hoc: Compute achieved power
**Inputs**		
	Tail(s)=Two	Effect size *f*=0.0708881
	Effect size *d*=0.3326908	α err prob=0.05
	α err prob=0.05	Total sample size=203
	Sample size group 1=157	Number of groups=2
	Sample size group 2=45	Number of measurements=2
		Correlation among repeated measures=0.257
		Nonsphericity correction ε=1
**Output**		
	Noncentrality parameter δ=1.9675292	Noncentrality parameter λ=2.7458948
	Critical *t*=1.9718962	Critical *F*=3.8881392
	df=200	Numerator df=1.0000000
	Power (1-β err prob)=0.4992920	Denominator df=201
		Power (1-β err prob)=0.3781274

^a^PHQ: Patient Health Questionnaire

## Discussion

### Principal Findings

Given the paucity of controlled trials of supplementing psychotherapy with text messaging, we utilized a historical control group to explore the impact of adding individually tailored between-session text messages to telephone-based psychotherapy, delivered as part of a post-ED support service. Working directly with therapists, we developed a text messaging intervention that emphasized collaboration between therapist and consumer in composing and scheduling between-session text messages related directly to the content of sessions. With the assistance of an IT partner (goACT), the text messaging intervention was implemented, with few practical issues, into an existing service.

To our knowledge, this is one of the first controlled studies of adding SMS, or text messaging, to psychotherapy. The study had high quality pre-post data because of the service’s session-by-session assessment protocol. The text messaging intervention was designed and implemented rapidly in practice, with informal feedback from clinicians and consumers indicating it was quick to learn, easy to implement, and a worthwhile addition to therapy. We anticipate the intervention could be transported easily to other clinical settings.

Intervention and control groups both showed significant improvements in depression, anxiety, and functional impairment over time indicating positive impacts of the IAPT@Flinders program. In fact, using ‘reliable change’ criteria set out by Gyani and colleagues [36], we found 76% of our sample demonstrated significant positive improvements, compared with 63.7% reported in the first year of the UK IAPT program. As such, the standard care received by both groups had a strong positive effect on psychological symptoms.

### Limitations

Consumers receiving the text messaging intervention in addition to standard care did not show significantly better clinical outcomes than a historical control group who received standard care without text messaging. A number of factors should be considered in interpreting this finding. First, we sought to detect the additional benefit of adding text messaging to what was already an efficacious treatment (low intensity telephone-based CBT) that was generating significant improvements in psychological symptoms. In contrast, other published trials have tested interventions against less active control groups that showed little change over time. Second, post-hoc power analyses suggested the study was underpowered to detect statistically significant effects of the size noted. Thus, genuine differences between the groups may have been obscured by lack of power. Third, the use of a historical control group, while a pragmatic solution for a counterfactual, raises some concerns about the comparability of the two groups and the causes of any observed differences. For example, the fewer number of sessions noted in the treatment group may be due to improvements in efficiency in the service over time.

With these limitations in mind, we provide the following suggestions for boosting the impact of the intervention and the power of the study design. The messages in the current study emphasized two core behavior change principles, namely, psychoeducation and homework/goal reminders. However, Michie and colleagues [37] have identified 93 behavior change techniques used in behavior change interventions; thus, many different behavior change techniques exist that were not utilized in the current study. The addition of other message types (eg, reward messages for goal completion, messages inviting consumers to monitor mood) may help supplement additional psychotherapy processes. Second, text messages in the current trial were one-way only. Providing consumers with the opportunity to reply to messages (including rating their usefulness, notifying of goal completion) could increase consumer engagement with the messages and alert therapists to which messages are the most engaging. Finally, while SMS text messaging remains the dominant messaging platform, modern smartphones have access to multiple messaging platforms (eg, Whatsapp, Google hangouts, iMessage), which have additional features such as multimedia and emoticons that could enhance the richness of messages being sent to consumers.

Future evaluations of this type of intervention should aspire to be randomized controlled trials, although these can be difficult to implement in clinical practice due to limited resources, lack of personnel, risk of consumer disengagement, and contamination. An alternative is a design similar to that of the current study, but with some refinements: larger sample, structured follow-up, detailed attendance records, the use of multiple therapists, the collection of detailed intake information (including mobile phone ownership and use), and statistical techniques such as propensity score matching to improve matching of intervention and historical control participants. We also recommend the implementation of a session-by-session assessment protocol, which is both clinically useful [38] and also ensures at least two data points for the majority of individuals receiving a service.

### Conclusions

In conclusion, the addition of individually tailored between-session text messaging to consumers receiving telephone-based psychotherapy following an ED admission, did not significantly improve clinical outcomes. However, this finding should be treated with caution given study design and power. The text messaging intervention was easy to implement and received positive feedback from therapists and consumers. For future trials, we specifically recommend refinement of the text messaging protocol as well as a randomized controlled trial study design to investigate whether the addition of mobile phone-based intervention components to psychotherapy can enhance consumer outcomes.

## Abbreviations

ANOVA: analysis of variance
CBT: cognitive behavioral therapy
DNA: did not attend
ED: emergency department
GAD-7: Generalized Anxiety Disorder questionnaire
IAPT: Improving Access to Psychological Therapies
PHQ-9: Patient Health Questionnaire
SMS: short message service
WSAS: Work and Social Adjustment Scale
